# Programmable SERS active substrates for chemical and biosensing applications using amorphous/crystalline hybrid silicon nanomaterial

**DOI:** 10.1038/srep19663

**Published:** 2016-01-20

**Authors:** Jeffery Alexander Powell, Krishnan Venkatakrishnan, Bo Tan

**Affiliations:** 1 Ultrashort laser nanomanufacturing research facility, Department of Mechanical and Industrial Engineering, Ryerson University, 350 Victoria Street, Toronto, ON, M5B 2K3, Canada; 2 Nano-imaging lab, Department of Aerospace Engineering, Ryerson University, 350 Victoria Street, Toronto, ON, M5B 2K3, Canada

## Abstract

We present the creation of a unique nanostructured amorphous/crystalline hybrid silicon material that exhibits surface enhanced Raman scattering (SERS) activity. This nanomaterial is an interconnected network of amorphous/crystalline nanospheroids which form a nanoweb structure; to our knowledge this material has not been previously observed nor has it been applied for use as a SERS sensing material. This material is formed using a femtosecond synthesis technique which facilitates a laser plume ion condensation formation mechanism. By fine-tuning the laser plume temperature and ion interaction mechanisms within the plume, we are able to precisely program the relative proportion of crystalline Si to amorphous Si content in the nanospheroids as well as the size distribution of individual nanospheroids and the size of Raman hotspot nanogaps. With the use of Rhodamine 6G (R6G) and Crystal Violet (CV) chemical dyes, we have been able to observe a maximum enhancement factor of 5.38 × 10^6^ and 3.72 × 10^6^ respectively, for the hybrid nanomaterial compared to a bulk Si wafer substrate. With the creation of a silicon-based nanomaterial capable of SERS detection of analytes, this work demonstrates a redefinition of the role of nanostructured Si from an inactive to SERS active role in nano-Raman sensing applications.

The field of nano-Raman sensing is growing year by year with further advancements to enhancement of well-established sensing materials^1^,^2^. Raman scattering is an important technique in the field of chemical and bio-sensing because it offers the ability to detect these analytes at single molecule concentrations^3^. Nano-Raman techniques also have the ability to detect a wide breadth of analytes; not only can nano-Raman device detect various chemicals including, pollutants in water supplies^4^, explosive chemicals^5^, and for pharmaceutical chemical identification^6^, nano-Raman has the ability to be used a sensing technique for many biomolecules as well. Nano-Raman sensors also have the capability to detect various cancer cells^7^,^8^, bacteria^9^, RNA and DNA^10^,^11^, and viruses^12^.

The primary issue nano-Raman scattering observation is the intensity of the scattered signal; only 10^−12^ incident photons^13^ will inelastically scatter resulting in Raman characterization being an impractical tool without significant enhancement in signal. SERS enhancement serves as a main focus for current research as nano-Raman sensing due to the extremely high enhancement factor and the exceptional sensitivity that SERS activation can achieve. It has been established that the main source of SERS enhancement for metallic nanomaterials comes from a phenomenon known as surface plasmon resonance (SPR); which is the collective oscillation of electrons about atomic nuclei caused by incident electromagnetic radiation. The increase in sensitivity to an analyte originates from the enhancement of electric field caused by SPR which is transferred to an analyte molecule resulting in a larger cross-section of Raman scattered photons^14^. Noble metals (gold, silver, etc.) have been predominantly used as SERS materials since they have a well-established property of exhibiting surface plasmon resonance (SPR) in the visible and near-infrared (NIR) spectrum, the wavelength range for most Raman lasers^15^. While SPR is the major source of Raman enhancement, it alone cannot provide the tremendous observed enhancement factors of these nanostructured materials; the physical morphology plays an important role as well and works in concert with SPR. Many different nanostructures have been created and have demonstrated enhanced Raman scattering, including nanostars^16^, nanofilms on arranged arrays of nanowires^17^, hollow or solid nanocubes^18^ or nanoshells^19^. The common origin of enhancement for most of these nanostructures is the creation of localized regions of concentrated electromagnetic field, which are known as Raman or plasmonic hotspots. Hot spots usually form in the nanogaps between two nanostructures^20^, the simplest being the gap between two nanoparticles^15^ but can be formed between nanostructures of many types^21^; this means however, that the creation of nanogaps is highly dependent on the nanostructure size, the wavelength of Raman laser and the analyte molecule size^13^, but with precise control single molecule detection is possible^3^.

There is however, a significant lack of research focus on developing materials that utilize semiconductor based nanomaterials, specifically silicon, for use as SERS enhancement substrates. This is due to nanostructured silicon materials being relatively dormant in terms of direct SERS activation when fabricated using conventional synthesis techniques. For example Wells *et al.* have achieved an EF value of 510 for the detection of Zn phthalocyanine on a Si nanopillar array fabricated using nanolithography techniques^22^. Other researchers have also reported EF values 8–28^23^, 7.1–70^24^, and 10^3^ 25. While substantial achievements in Si-based SERS activation, these EF values are orders of magnitude lower than the EF values reported from noble metal nanostructures; for example Garcia-Leis *et al.*^26^ have reported an EF value of 1.72 × 10^5^ for Ag nanostars, Wang *et al.*^27^ have reported a maximum EF value of 10^7^ for gold nanoparticle arrays and Tao *et al.*^28^ have reported an EF value of 2 × 10^9^ for Ag nanowire monolayers. As such, of the currently researched SERS nanomaterials, nanostructured silicon is employed primarily as a scaffold or substrate for noble metal Raman active nanostructures^29^,^30^,^31^; nanostructured silicon does not play a major active role in Raman enhancement. There is a significant incentive to fill this void of SERS active silicon nanomaterials because silicon is the building block material for all existing electronic devices and having access to a Raman active nanostructured silicon material would not require the development of new integration techniques for creating Raman sensing devices. A sizable amount of research has been conducted to investigate the Raman activity of semiconductor nanomaterials yielding promising results. SERS enhancement has been observed in ZnO^32^, ZnS^33^, CdS^34^ and CuO^35^, each in colloidal suspension. Competitive EF values, on the order of 10^6^ have also been observed in three dimensional TiO_2_ nanostructures^36^,^37^,^38^,^39^,^40^,^41^,^42^,^43^,^44^,^45^,^46^. These semiconductor materials and to a greater extent silicon, are more attractive as SERS materials due to the breadth of controllability over material properties (including band gap, dopants, physical morphology, stoichiometry, phase crystallinity, nanostructure size distributions etc.^40^) that semiconductors have over metals. Therefore a highly SERS active Si nanomaterial opens up new opportunities to use as a SERS sensing substrate rather than cost-prohibitive, difficult to fabricate noble metal SERS nanostructures that lack depth of control over material properties and physical morphology. The use of Si based SERS nanomaterials could lead to the proliferation of economical SERS detection devices for a vast array of applications.

With our current research we observe a new phenomenon for SERS activation using nanostructured Si that we have precise control over with the formation mechanism that allows us to create a SERS active material from a dormant Raman Si wafer material. In this study, we have created an entirely new type of nanomaterial which is as to yet, not producible by other methods; we are able to create a hybrid crystalline/amorphous Si nanospheroids within a laser ionization plume, which fuse and deposit onto the silicon substrate as an interconnected nanoweb network. Rather than individual crystalline and amorphous nanoparticles, with the ultrafast femtosecond laser synthesis we able to create nanospheroids that have regimes of crystalline and amorphous silicon throughout individual nanospheroids. By altering the ionization mechanisms and temperature within the laser plume, we are able to precisely control the structural arrangement of the silicon ions to form this hybrid silicon nanomaterial. Not only are we able to program the crystalline/amorphous content of the individual nanospheroids, we can manipulate the morphology of the nanospheroids and how they will arrange themselves when deposited on the substrate surface. Due to this unique particle formation mechanism, these formed nanospheroids are distinctive in their structural character which allows them to be much more SERS active than bulk single crystal Si due to a higher concentration of grain boundaries within the nanospheroids. This combination of amorphous/crystalline grains within individual nanospheroids of an interconnected nanospheroid network is an entirely new concept for nano-Raman activation. Figure 1 is an overall schematic and SERS enhancement figures for molecular dyes on Si nanoweb strucutres.

Our goal with this research is to demonstrate that we able to synthesize an entirely new silicon nanomaterial has SERS enhancing properties not present in bulk silicon material or other nanostructured Si materials and that we can obtain precisely control the production of this unique nanomaterial which is unattainable with other nanomaterial formation mechanisms. In addition, we demonstrate the viability of a Si Raman active nanomaterial as a chemical sensing substrate without the need for traditional noble metal SERS activation.

## Experimental Procedure

### Laser Ablation/Raman analysis/Material characterization

A pulsed Yb-doped fibre amplified femtosecond laser was used to fabricate the silicon nanostructures. In order to maximize the control over the nanoweb fabrication, some of the parameters of the laser need to be fixed, in this experiment the laser wavelength (1030 nm), polarization (circular) and the laser power (16 W) were fixed. The parameters that were varied were the repetition rate, dwell time and pulse width.

The nanostructures were created on a 5 × 5 mm array of points with a 50 μm point spacing; the array was plotted using EzCAD software and controlled using a piezo-driven raster scanner. The silicon substrates that were used were crystalline silicon wafers with orientation <110>.

Each ablation area was examined before and after a dye was coated onto the ablation using a B&W Tek, Inc NanoRam® handheld Raman system. The Raman excitation laser used has a wavelength 785 nm at a power of 350 mW. The bare nanofibre ablation areas were analyzed using the Raman laser to determine how the nanostructures change the Raman spectra compared to the unablated substrate in terms of the Raman intensity as well as any structural/compositional changes caused by the ablation. The dyes used to test the SERS enhancement factor of the silicon nanostructures were Rhodamine 6 G and Crystal Violet, which are popular dyes for SERS analysis due to their large Raman cross-section. To determine a sensitivity range for the Si nanoweb structures, each dye was coated onto individual ablation areas at two different concentrations, 8 × 10^−3^ M and 8 × 10^−6^ M. A single drop of each dye concentration for both dyes was applied to a separate ablation area prior to Raman analysis. Each resultant Raman spectra were obtain at 3 s collection time and repeated in triplicate then averaged.

To confirm the presence of and characterize the structure of the silicon nanoweb networks, high-resolution scanning electron microscopy (HRSEM) was used. SEM was used to determine the nanospheroid size distribution and nanogap size distribution for each ablation area. Using the HRSEM images of the nanoweb substrates and ImageJ image processing software, the average size of the nanospheroid was calculated. Using ImageJ, the particle size is calculated manually; first the scale is adjusted based on the magnification of the image then the spheroid size is measured from the outer diameter of each spheroid; the nanogap size distribution was measured using the same technique. To obtain cross-sectional images, substrates were split down the middle through an ablation area, and the nanofibres were imaged at an angle. Gold sputtering of the nanoweb was necessary because of excessive charging due to the fibres being composed of silicon.

High-resolution transmission electron microscopy (HRTEM) was also used to image and analyze the nanoweb and nanospheroid shape and size. HRTEM involves using a carbon grid to swab the sample to attach nanospheroid clusters to the grid and then the grid is scanned. Using the fast Fourier transform (FFT) analysis we able to determine the crystal orientations present within each nanospheroid.

X-ray diffraction was used to analyze the composition of the nanostructures as well as the relative proportion of crystalline and amorphous content of the nanostructures. The XRD data was collected using a Bruker AXS D8 Advance microdiffraction system equipped with a Cu-K source and graphite monochromator to eliminate unwanted Cu-K-beta lines. In order to obtain the relative proportion of crystalline to amorphous content, the XRD data underwent Reitveld analysis.

## Results and Discussion

### Hybrid Nanospheroid formation

**Figure 2:**
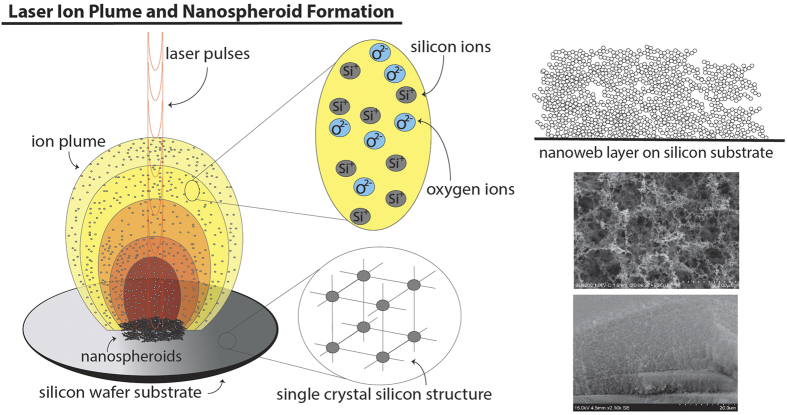
Schematic representation of ion plume nanospheroid formation mechanism.

**Figure 3:**
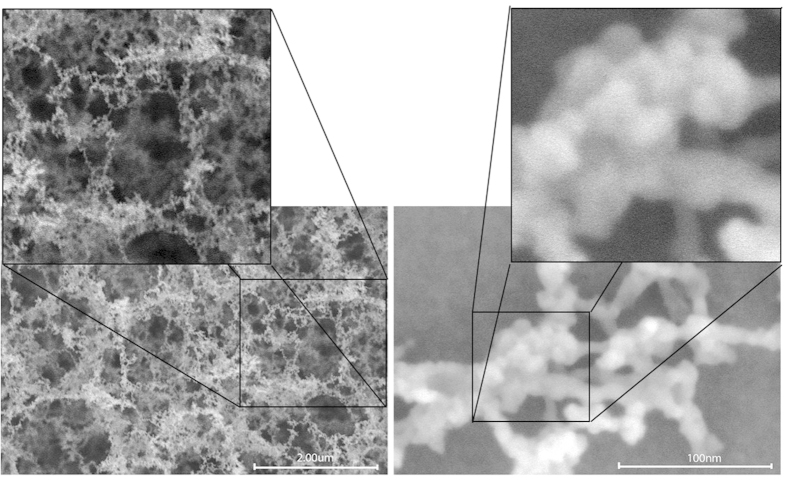
HRSEM images of hybrid amorphous/crystalline silicon nanoweb structures.

The unique nanomaterial that we have been able to create with the ultrafast femtosecond laser is a material that, to our knowledge, cannot be formed using any other fabrication technique; lasers with longer pulse widths (nanosecond, picosecond, etc.) are unable to create this material because only a laser with femtosecond pulse width is able to cause such high temperatures that when the pulses strike the silicon surface, Si atoms are immediately ionized and form an ion plume above the silicon surface without the loss of energy to heating of the substrate^41^. Figure 2 is schematic showing the laser ion plume formation mechanism with SEM images of formed nanoweb structures. The nanomaterial that we have observed is an interconnected network of hybrid amorphous/crystalline nanospheroids to form a nanoweb structure. Figure 3 shows the nanoweb structure and the interconnected nanospheroid nature of the nanoweb material.

**Figure 4:**
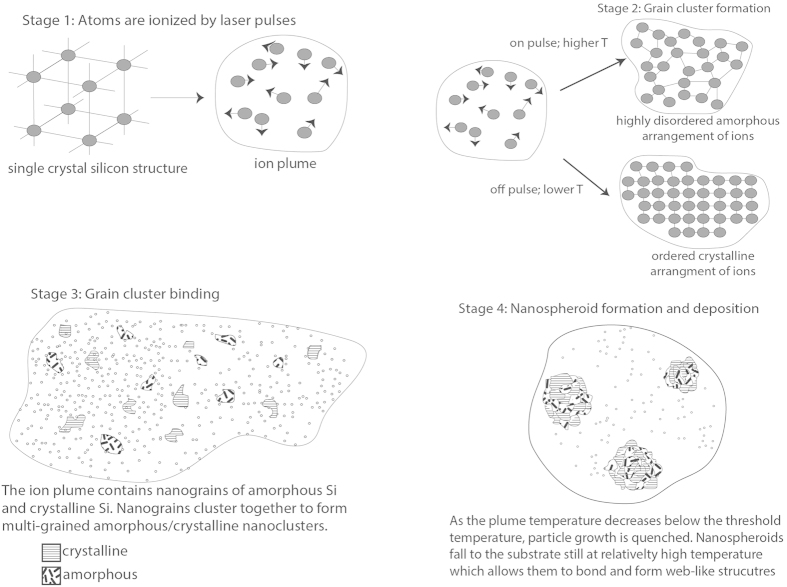
Schematic representation of theoretical hybrid amorphous/crystalline nanospheroid formation mechanism.

We have theorized that this hybrid of crystalline and amorphous phases within individual nanospheroids is due to the rapid fluctuation of temperature of the plume caused by the laser pulses. The plume temperature is quickly shifting creating moments of higher temperature and relatively lower temperature^42^. At higher temperatures, the Si ions when interacting are more likely to arrange themselves into amorphous structures because they will have higher thermal energy and as such will behave in a less organized fashion^43^, resulting in a more random assembly. Between pulses however, the plume temperature will be lower^42^ because the laser is not providing additional thermal energy, therefore the Si ions will be less energetic and assemble themselves into a more organized and lower energy arrangement, resulting in a crystalline structure. As time passes, these Si atoms that have formed amorphous or crystalline arrangements will collide and bind together to form random arrangements of amorphous and crystalline nanograins. These will then coalesce and deposit themselves onto the substrate surface as nanospheroids that have regions, within the same spheroid, of amorphous and crystalline silicon. Figure 4 is a schematic diagram of this process.

**Figure 5:**
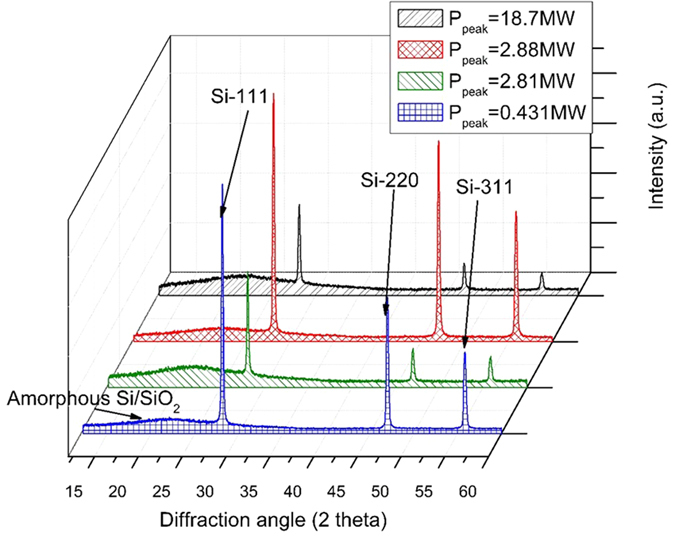
XRD Spectra for nanoweb structures.

The XRD results (Fig. 5) show that within our nanoweb samples we have observed the presence of both crystalline and amorphous Si and amorphous SiO_2_. These spectra also show three sharp peaks indicating the presence of multiple orientations of crystalline Si. There is a sharp peak for single crystal Si from the {111} plane for each sample and sharp peaks for the {220} and {311} planes. The existence of these multiple orientations of crystalline Si supports our hypothesis that we are creating nanospheroids with randomly oriented grains of Si. These peaks are present for each XRD spectra in Fig. 5; therefore regardless of plume conditions we are able to create nanospheroids with randomly oriented Si grains.

The other major observation from the XRD spectra is that each nanoweb layer has a significant portion of amorphous content, from which we can surmise that in addition to the multiple crystallographic orientations of Si present in each particle, amorphization of silicon is occurring during the particle formation process as well as an oxidation process which forms amorphous SiO_2_^43^. This coincides with the proposed mechanism, in which the individual nanospheroids consist of Si grains with different crystallographic orientations and amorphous Si grains.

**Figure 6:**
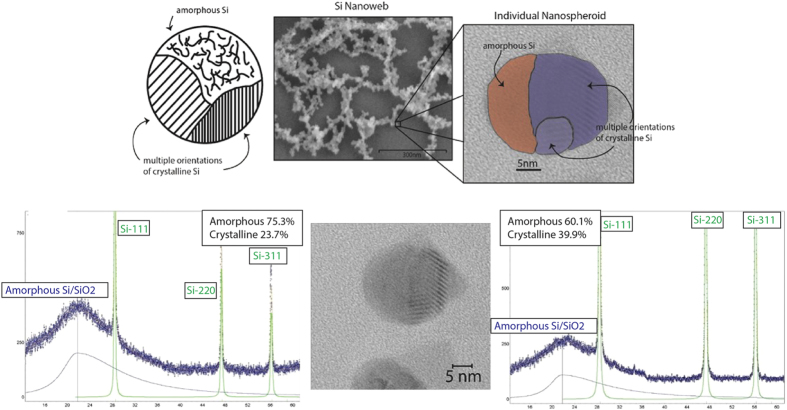
A Schematic showing hybrid nature of nanospheroids with HRSEM and HRTEM images showing nanoweb structure and amorphous/crystalline grains within nanospheroid and Rietveld spectra for nanoweb created at high peak power (left) and lower peak power (right).

Using Reitveld analysis we have determined what proportion of crystalline to amorphous material is present (Fig. 6). Reitveld analysis spectra for 214 fs at 4 MHz are not included due to equivalent sample compositions between 214 fs and 1428 fs at 4 MHz. The sample spectra for 1478 fs at 26 MHz are not included for the same reason. The Reitveld analysis shows that at 214 fs the sample is 76% amorphous and 24% crystalline and at 1428 fs the sample is 40% amorphous and 60% crystalline.

With the level of control that we have over the conditions within the Si ion plume, we are able to influence the particle formation mechanism in order to construct a selected physical morphology of the deposited nanoweb layer. The two aspects of the physical morphology that are highlighted in this research are the diameter of the deposited hybrid nanospheroids and the thickness of the nanoweb layer on the substrate surface. To design the morphology of the nanoweb layer we have modified how the laser interacts with the substrate surface; we have control over the time interval of each laser pulse, known as the pulse width, the time between the laser pulses, known as the repetition rate and the time spent ablating each ablation point known as the dwell time.

As with the hybrid structure formation, controlling the morphology of the individual nanospheroids, specifically the diameter of the nanospheroids, is dependent on the temperature within the ion plume. At high plume temperatures, enough energy is supplied to the nanograins so that they can continue to grow and bind with other nanograins^44^, and thus increase in size. However, when the temperature of the plume decreases below a certain point the growth rate of the grains is inhibited, effectively quenching grain growth^41^ and initiates the nanospheroids formation stage and defining the particle size. Since we can use the laser attributes to define the plume temperature, we can have direct control over the diameter of the nanospheroids.

This change in particle size is due to the effects of the repetition rate and the pulse width on the temperature within the ion plume in which the particles are forged. By changing the repetition rate, we are changing the number of pulses that strike the sample surface every second. By changing the pulse width we are changing the time between laser pulses striking the substrate. Both of these laser parameters will affect the peak power of the laser pulses and thus the energy of the pulses. Peak power and pulse energy are calculated using the following two equations:


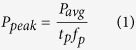



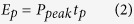


where P_peak_ is the peak power P_avg_ is the average power, t_p_ is the pulse width, f_p_ is the repetition rate and E_p_ is the pulse energy

At higher energy, it is expected that the plume will have a higher overall temperature, due to the fact that more energy can be transferred to the substrate thus allow for larger excitation of phonons. When the average plume temperature is high, the growth rate of the nanoparticles is high resulting in larger particles at higher plume temperatures. This is due to the temperature dependence of particle growth rates. Particle growth is expressed by the following equation:


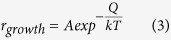


where r_growth_ is the growth rate, A is an independent coefficient, Q is the activation energy required to initiate particle growth, k is Boltzmann’s constant, and T is the temperature^47^. As the temperature of the system increases the growth rate will have an exponential increase, therefore by increasing the peak power, we are transferring more energy to the substrate and the laser plume thus increasing the plume temperature and increasing the growth rate and the size of the formed particles. As a result, both the repetition rate and the pulse width of the laser pulses will have a significant effect on the particle size.

These results show that we can precisely control the material structure of the individual nanospheroids as well as the morphology of the nanospheroids and nanoweb layer by manipulating the interactions between the ultrafast laser pulses and the substrate material. As such we have direct regulation over the spheroid formation mechanism within the ion plume. The Raman characteristics of the formed nanoweb layer are dependent on several different sources each of which we can manipulate by controlling the nanospheroid formation mechanism.

Another of the critical features of the formation mechanism that we have proposed is the presence of polycrystalline grains and amorphous grains within individual nanospheroids. To confirm the crystalline/amorphous grains in the nanospheroids we have taken TEM images of dispersed nanospheroids to determine crystal orientations and amorphous regions.

### SERS Enhancement from hybrid nanostructures

The SERS and Raman enhancement from our unique nanomaterial is two-fold; the hybrid structure of the individual nanospheroids, as well as the physical morphology of the nanospheroids on the substrate surface contributes to the detection of the analyte. The sum of these contributions leads to the observed enhancement of the Raman signal of the R6 G dye analyte.

#### Raman Enhancement from hybrid nanostructures

The SERS and Raman enhancement from our unique nanomaterial is two-fold; the hybrid structure of the individual nanospheroids, as well as the physical morphology of the nanospheroids on the substrate surface contributes to the detection of the analyte. The sum of these contributions leads to the observed enhancement of the Raman signal of the silicon or any deposited analyte. To quantify this enhancement, the following equation was used^48^.


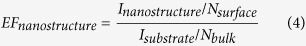


where 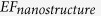
is a proportionality that quantifies the Raman enhancement between the substrate and the nanostructures by comparing the intensity of the peaks at a characteristic Raman shift of the analyte for both the substrate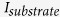 and the nanostructured surface 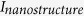. The 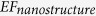 proportionality requires a factor that takes into account the interaction volume of the Raman laser and the number of dye molecules within this volume which contribute to Raman enhancement; these factors are 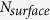 and 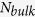 for the nanostructures and substrate, respectively. 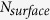 takes into account the effective surface area of the nanostructures and the absorption of dye into the nanoweb structure within the Raman interaction volume. Some assumptions were made when calculating 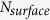 and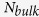; the density of the nanostructures is assumed to be identical to crystalline silicon, the dye absorption and surface area are assumed to be similar to the values established by Maznichenko *et al.*^46^ due to the same ion plume formation mechanism is employed to create both the TiO2 and hybrid Si nanostructures.

#### Raman Results for bare substrates

**Figure 7:**
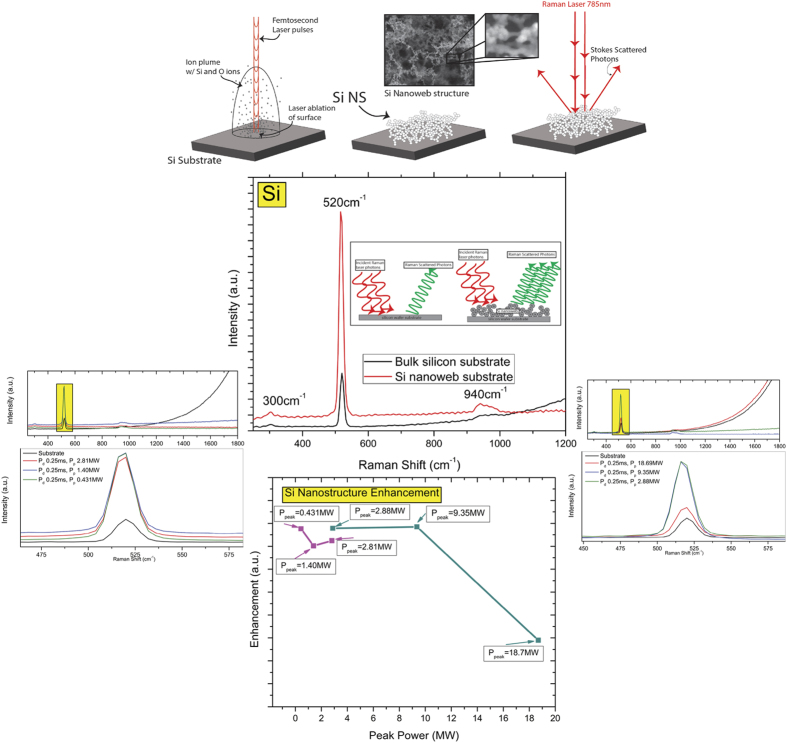
Schematic of Raman enhancement of bare Si nanowebs, Raman spectra silicon peaks on Si nanoweb and Si wafer substrate, Raman spectra 520 cm^−1^ for Si on Si nanowebs, and EF values at 520 cm^−1^ for Si on Si nanowebs as a function of Peak Power.

A schematic of this portion of the experiment, the Raman spectra of nanoweb structures compared to a bulk Si wafer substrate spectra, the Raman spectra of each nanoweb structure with zoomed spectra for the 520 cm^−1^ Si peak, and the enhancement factors for each substrate are demonstrated in Fig. 7.

#### Raman Results for dye-coated substrates

**Figure 8:**
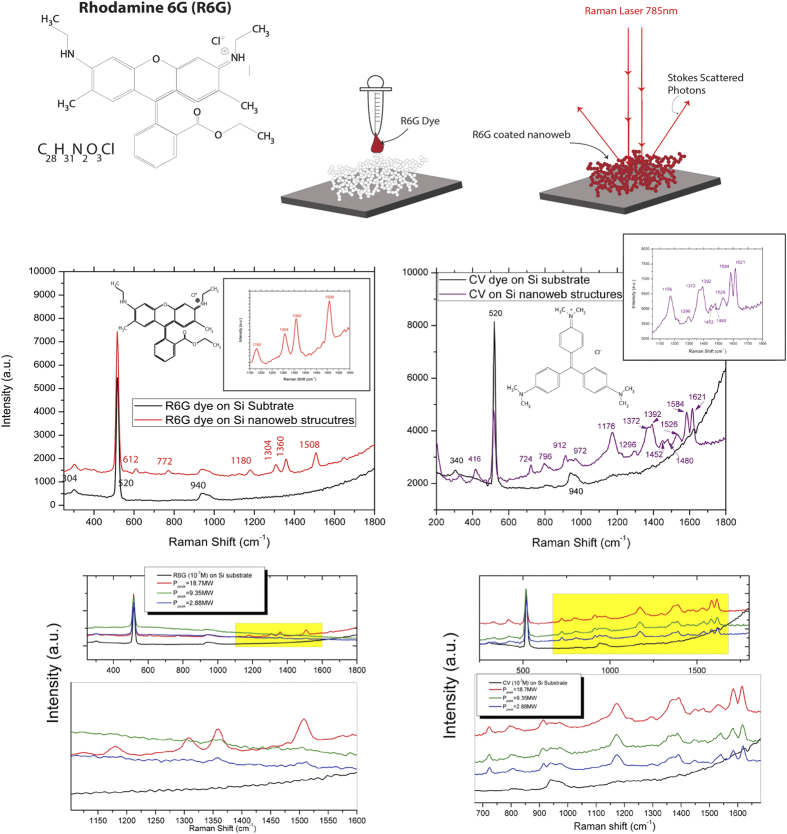
Schematic of Raman enhancement sensing with a molecular dye on Si nanowebs, Raman spectra of R6 G and CV dye on Si nanoweb and Si wafer substrate, Raman spectra for R6 G and CV on Si nanowebs for each laser ion plume condition.

A schematic of this portion of the experiment with a molecular dye, the Raman spectra of the dye-coated substrates, the Raman spectra of each nanoweb structure with zoomed spectra for the 1360 cm^−1^ R6 G peak and for the 1621 cm^−1 ^CV peak at both 10^−3^ M and 10^−6^ M concentrations, and the enhancement factors as function of peak power for each concentration on the nanoweb substrates are shown for both R6 G (@ 1360 cm^−1^) and CV (@ 1621 cm^−1^) in Fig. 8.

**Figure 9:**
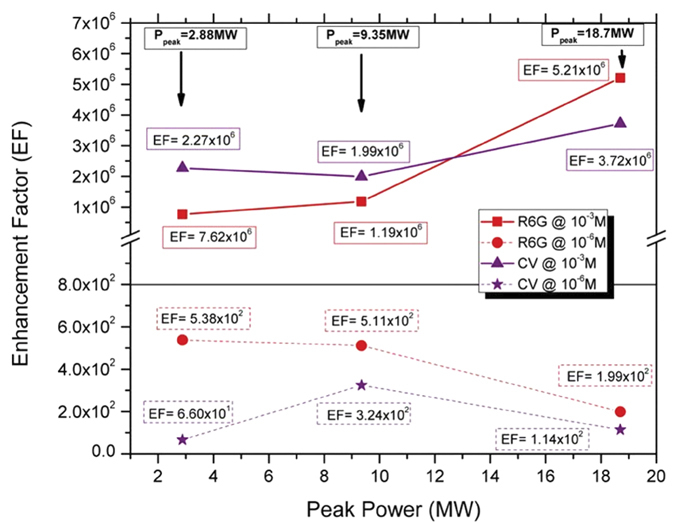
EF values for R6 G and CV dyes on Si nanoweb structures at 10^−3^ M and 10^−6^ M concentrations as a function of peak power.

From the Raman spectra and EF values (Figs 8 and 9), we have observed a significant enhancement in intensity of the characteristic Raman peaks of both dyes on the nanoweb layer substrates compared to dyes on the Si substrate. When coated with the either the R6 G dye or the CV dye, we have observed that the bulk Si substrate spectra has no response to the presence of a dye; however when a dye is coated onto the nanoweb layer substrates, the characteristic peaks of the associated dye are clear well-defined. We observe a maximum enhancement of 5.21 × 10^6^ and 3.72 × 10^6^ for both R6 G and CV dyes respectively at 10^−3^ M concentration at a peak power of 18.7 MW. For 10^−6^ M concentration of both R6 G and CV the EF values while several orders of magnitude lower than the EF values at higher dye concentration, the Si nanoweb structures there remains observable enhancement of each dye spectra. This lends credence to the theory that our new material is highly Raman active and is able to detect an analyte much more readily than bulk silicon. While it has been proven that roughened noble metal substrates exhibit SERS enhancement^49^ compared to noble smoother substrates, these results show that our Si nanoweb structures alone are able to enhance the detection of a molecular dye without the aid of noble metal SERS enhancement.

## Semiconductor SERS enhancement

Due to current theoretical advances, our observed SERS enhancement can be attributed to a series of linked resonances only possible with semiconductor nanomaterials^32^,^33^,^34^,^35^. The resonances that work in tandem to achieve our observed EF are surface plasmon resonance, molecular resonance, charge-transfer resonance and exciton resonance.

### SPR contributors

#### Hybrid Structure enhancement

**Figure 10:**
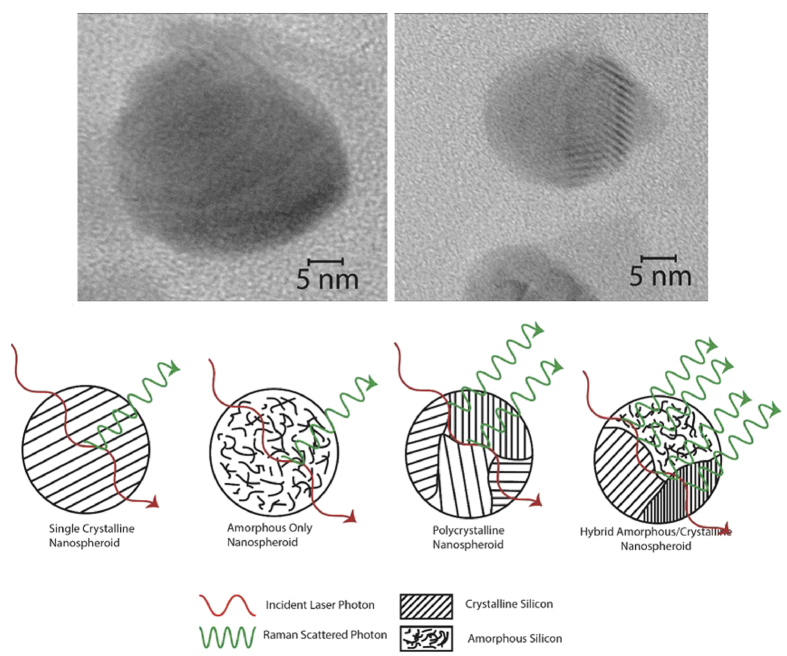
HRTEM images of Hybrid Amorphous/Crystalline nanosperoids, and a schematic of Grain Boundary Raman Scattering within a Single Crystalline Nanosperoid, an Amorphous Only Nanospheroid, a Polycrystalline Nanospheroid and a Hybrid Amorphous/Crystalline Nanosperoid.

The amorphous/crystalline hybrid structure of the individual nanospheroids provides a distinct source of Raman enhancement due to its unordinary structure. The hybrid nanograin structure of the nanospheroids results in a high concentration of grain boundaries between grains with different orientations of crystalline silicon ([111], [220], [311]) and grain boundaries between crystalline silicon grains and gains of amorphous silicon within each nanospheroid. With TEM imaging, FFT analysis and XRD spectra, we have observed the presence of grains of multiple orientations of crystalline silicon and amorphous silicon in each nanospheroid. The grain boundary concentration affects how the light from the Raman laser scatters within the nanospheroid structure. Veprek *et al.,* have demonstrated that grain boundaries in nanocrystalline silicon films exhibit enhancement in Raman scattering intensity, but is limited to processes that involve coupling of the electromagnetic field via charge density fluctuations in the grain boundaries to the bulk of the crystallites^50^. Due to the bond stretching and compression (bond dilation) that occurs at the grain boundaries, a local electric dipole moment is formed which results in enhanced coupling with the EM field. Therefore with larger grain boundary concentration, the stronger the coupling with the EM fields thus more Raman enhancement. At higher peak power, we observe a greater amount of amorphous content and with this we can infer a greater concentration of grain boundaries (Fig. 10); the nanoweb substrates created at higher peak power have the largest observed Raman enhancement of all our formed nanoweb structures.

#### Nanospheroid Size and Nanogap Enhancement

**Figure 11:**
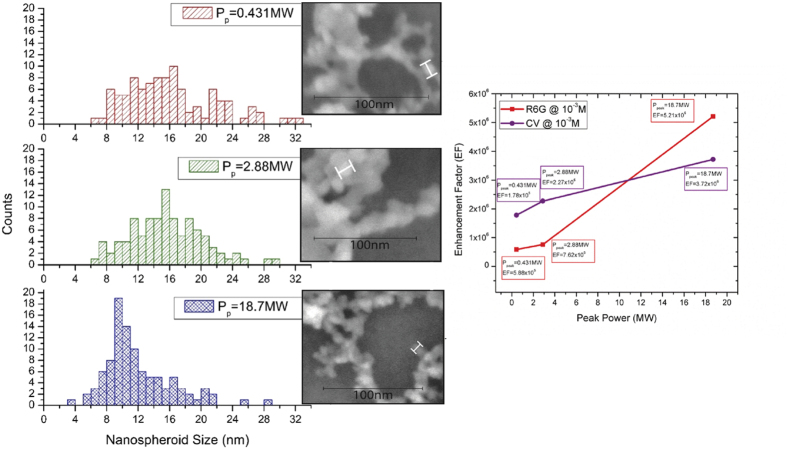
Nanospheroid size distribution and Enhancement Factor as a function of peak power at 0.431 MW, 2.88 MW and 18.7 MW.

The material structure of the nanospheroids is not the only source of Raman enhancement for our material, the nanospheroid size and the assembly of nanospheroids on the substrate surface provide enhancement as well. By changing the laser parameters to control the peak power, we are able to adjust the spheroid size distribution of the silicon nanospheroids within the nanoweb. Fig. 11 shows the nanospheroid size distributions for different nanoweb structures.

These figures show that as the peak power decreases, the distribution of nanospheroid sizes broadens and the median value shifts to larger spheroid size. This result is congruent with our knowledge of the femtosecond lasers effect on the plume temperature, and the condensation and grain growth of nanostructures. The Raman spectra enhancement factors for the R6 G dye and CV dyes on the silicon nanowebs (Fig. 11) indicate that as we increase the distribution of nanospheroid size and effectively create a larger volume of large nanospheroids the enhancement of the dye peaks diminishes; therefore at large peak power, we create larger nanospheroids which cause greater enhancement. This correlation however requires further study because not only do the nanospheroids decrease as peak power increases but the nanogap size decreases as well (Fig 11).

#### Nanogap/Raman hot-spot enhancement

It has been shown and well established, that the size of the nanogaps between Raman active nanostructures has a direct and crucial influence on the Raman enhancement factor of a material^44^,^50^.

**Figure 12:**
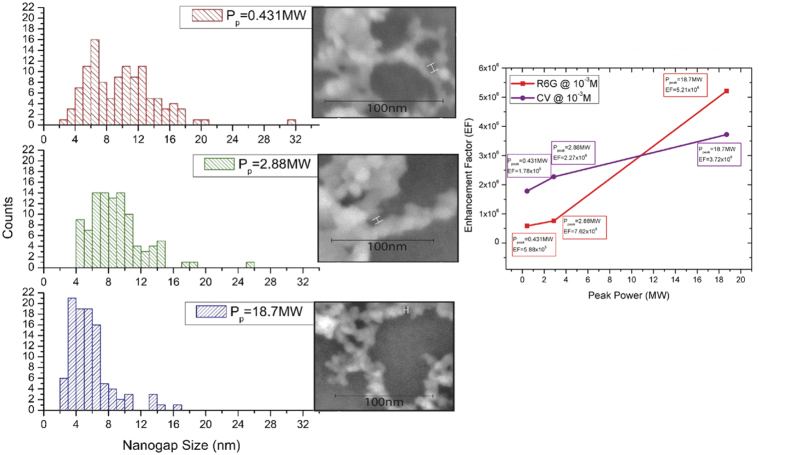
Nanogap size distribution and Enhancement Factor as a function of peak power for nanowebs created at 0.431 MW, 2.88 MW and 18.7 MW.

The unique structure of the nanoweb layer adds additional enhancement effects due to the formation of nanogaps between individual nanospheroids. These nanogaps are formed when the Si nanospheroids fuse together on the substrate surface to create fibre-like structures. These nanogaps allow for a concentration of electromagnetic field which allows for an amplification of the SERS enhancement^51^. It has been shown that when the nanogap size is significantly reduced, an order of magnitude enhancement is observed^20^,^43^,^52^. We have measured the nanogaps for three different nanoweb layers each created at different peak powers, the nanogap size distributions are displayed in Fig. 12.

These distributions clearly show that as the peak power is reduced the nanogaps between the nanospheroids increase in size and become less uniform. This increase in size correlates to a decrease in Raman enhancement seen in Fig. 12. The maximum EF is obtained at a peak power of 18.7 MW and the median nanogap size observed with this specific nanoweb is approximately 5.15 nm. While this value is dissimilar to reported optimal nanogap sizes^53^ for other SERS active nanomaterials^51^,^52^,^53^,^54^,^55^,^56^,^57^,^58^, this hybrid nanoweb material has a distinction in material character and a contribution to EF by the grain boundary scattering of the individual nanospheroids, which divorces currently established values for optimal nanogap size for noble metal nanostructures from our truly silicon based Raman active material. The role of the nanogap distribution also plays an important role in the SERS enhancement^59^,^60^,^61^; the nanogap distribution observed in Fig. 12 shows clear evidence of a broadening of nanogap size as peak power increases. While our observed nanogap size cannot be held to a standardized value for other nanomaterials, the principle governing Raman enhancement from a narrow distribution of nanogap sizes^53^ can still be applied to our nanostructures. As observed from Fig. 12, the enhancement factor decreases as the breadth of the nanogap distribution increases. This is due to a lack of efficiency in Raman scattering from the nanogaps^48^ and a smaller concentration of nanogaps within the optimal range for our hybrid Si nanomaterial.

#### Molecular, Charge-Transfer, and Exciton Resonance Contributors

Lombardi and Birke^40^ have established a comprehensive theory to describe and predict the SERS scattering in semiconductor materials. Their theory states that observed SERS enhancement with semiconducting nanomaterials are not mainly attributed to SPR, as in the case of metallic nanomaterials, but also ascribed to linked resonances between molecular resonance, charge-transfer resonance and exciton resonance. Their theory also suggests that not only are semiconductors capable of achieve EF values comparable to those of noble metal nanostructures, but semiconductor nanomaterials have the capacity to attain single molecule sensitivity for chemical sensing applications.

The contribution to SERS enhancement from molecular and charge transfer resonance occurs when the valence and conduction bands of the semiconductor nanomaterial are similar in energy level to the lowest unoccupied molecular orbital (LUMO) and highest occupied molecular orbital (HOMO), respectively. When this similarity in energy level occurs, a charge transfer can occur between the valence band edge and the LUMO or the conduction band edge and the HOMO resulting in a SERS enhancement, the most intense of which occurs when transitions terminate at band edges. In addition exciton resonances occur when excitons (electron-hole pairs) created in the semiconductor nanomaterial by optical absorption. These excitons created form exciton levels from below the conduction band to above the valence band which forms a sequence in the absorption spectra. When the nanoparticle size decreases below the Bohr radius for the semiconductor material, the exciton levels diverge, due to quantum confinement, leading to a dependence of the SERS enhancement spectra on the semiconductor nanoparticle size. Each of the contributions to semiconductor SERS enhancement will be explored as research topic with the further development of these hybrid Si nanostructures.

## Conclusion

In this paper we have demonstrated the unique ability to create a Raman active nanomaterial from an inactive Raman bulk material. With this silicon based Raman active material, we are uniquely able to increase the cross-section of Raman scattered photons without the use of well-established SERS active nanomaterials. Not only are we able to activate the Raman sensitivity with our nanoweb material, we are able to precisely tune the Raman activity of this nanomaterial by controlling the ion plume formation mechanism, by controlling the laser/substrate interaction parameters. The material that we form is a nanoweb material composed of an interconnected network of hybrid amorphous/crystalline nanospheroids. The hybrid nature of the individual nanospheroids and the nanogap concentration of the nanoweb structure and the linked resonances present in this SERS active Si nanoweb semiconductor material are the major contributors to the increase in Raman activity. We have also proven that this nanomaterial has the potential to be used as a chemical sensing device with our observation of a maximum enhancement factor of 5.38 × 10^6^ for chemical dye R6 G and 3.72 × 10^6^ for CV dye. With these results, we have open up a new avenue for the use of silicon as a Raman active material for chemical sensing devices.

## Additional Information

**How to cite this article**: Powell, J. A. *et al.* Programmable SERS active substrates for chemical and biosensing applications using amorphous/crystalline hybrid silicon nanomaterial. *Sci. Rep.* **6**, 19663; doi: 10.1038/srep19663 (2016).

## Figures and Tables

**Figure 1:**
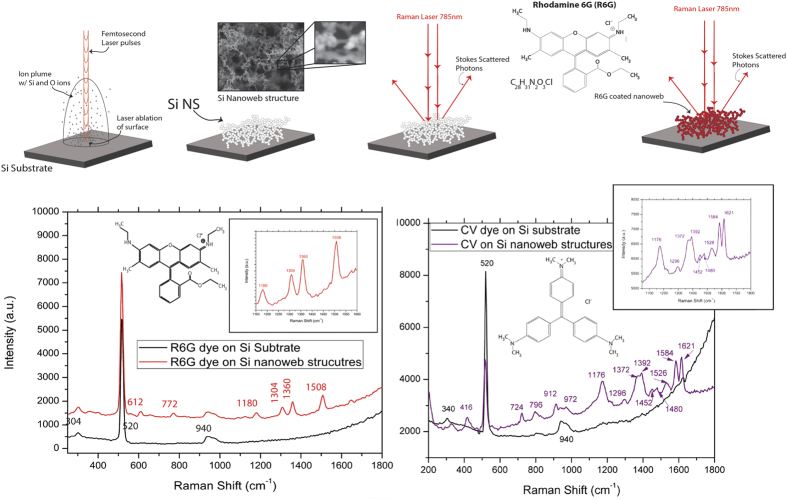
Overall schematic of Nanoweb formation and SERS enhancement of R6 G dye and CV dye.
